# Influence of psychiatric or social backgrounds on clinical decision making: a randomized, controlled multi-centre study

**DOI:** 10.1186/s12909-019-1897-z

**Published:** 2019-12-12

**Authors:** Yosuke Yamauchi, Takashi Shiga, Kiyoshi Shikino, Takahiro Uechi, Yasuaki Koyama, Nobuhiko Shimozawa, Eiji Hiraoka, Hiraku Funakoshi, Michiko Mizobe, Takahiro Imaizumi, Masatomi Ikusaka

**Affiliations:** 1 Department of Emergency and Critical Care Medicine, Tokyo Bay Urayasu/Ichikawa Medical Centre, 3-4-32 Todaijima, Urayasu, Chiba, Japan; 2 Department of General Medicine, University of Chiba Hospital, 1-8-1 Inohana, Chuo, Chiba, Chiba Japan; 3 Department of Emergency Medicine, International University of Health and Welfare, 537-3 Iguchi, Nasushiobara, Tochigi, Japan; 4 Department of General Medicine, Kyorin University School of Medicine, 6-20-2 Shinkawa, Mitaka, Tokyo, Japan; 5 Department of Emergency and Critical Care Medicine, University of Tsukuba Hospital, 2-1-1 Tenkubo, Tsukuba, Ibaragi, Japan; 6 Department of Emergency and Critical Care Medicine, St. Marianna University School of Medicine Hospital, 2-16-1 Sugao, Miyamae, Kawasaki, Kanagawa Japan; 7 Department of General Internal Medicine, Tokyo Bay Urayasu/Ichikawa Medical Centre, 3-4-32 Todaijima, Urayasu, Chiba, Japan; 8 Center for Advanced Medicine and Clinical Research, Nagoya University Hospital, 65 Tsurumai, Showa, Nagoya, Japan

**Keywords:** Clinical decision making, Cognitive bias, Case scenario

## Abstract

**Background:**

Frequent and repeated visits from patients with mental illness or free medical care recipients may elicit physicians’ negative emotions and influence their clinical decision making. This study investigated the impact of the psychiatric or social background of such patients on physicians’ decision making about whether to offer recommendations for further examinations and whether they expressed an appropriate disposition toward the patient.

**Methods:**

A randomized, controlled multi-centre study of residents in transitional, internal medicine, or emergency medicine was conducted in five hospitals. Upon randomization, participants were stratified by gender and postgraduate year, and they were allocated to scenario set 1 or 2. They answered questions pertaining to decision-making based on eight clinical vignettes. Half of the eight vignettes presented to scenario set 1 included additional patient information, such as that the patient had a past medical history of schizophrenia or that the patient was a recipient of free care who made frequent visits to the doctor (biased vignettes). The other half included no additional information (neutral vignettes). For scenario set 2, the four biased vignettes presented to scenario set 1 were neutralized, and the four neutral vignettes were rendered biased by providing additional information. After reading, participants answered decision-making questions regarding diagnostic examination, interventions, or patient disposition. The primary analysis was a repeated-measures ANOVA on the mean management accuracy score, with patient background information as a within-subject factor (no bias, free care recipients, or history of schizophrenia).

**Results:**

A total of 207 questionnaires were collected. Repeated-measures ANOVA showed that additional background information had influence on mean accuracy score (F(7, 206) = 13.84, *p* <  0.001 partial η2 = 0.063). Post hoc pairwise multiple comparison test, Sidak test, showed a significant difference between schizophrenia and no bias condition (*p* <  0.05). The ratings for patient likability were lower in the biased vignettes compared to the neutral vignettes, which was associated with the lower utilization of medical resources by the physicians.

**Conclusions:**

Additional background information on past medical history of schizophrenia increased physicians’ mistakes in decision making. Patients’ psychiatric backgrounds should not bias physicians’ decision-making. Based on these findings, physicians are recommended to avoid being influenced by medically unrelated information.

## Background

During clinical decision making, physicians attempt to be cordial and fair to every patient, and they believe that their decision making is not influenced by patients’ background information. However, in real world, it seems to be difficult for clinicians not to have emotional reactions toward patients [1–3].

Several studies have shown that physicians are not always rational and objective [4]. In primary care practice in the United Kingdom and the United States, female patients were found to have a lower chance of being examined for coronary heart disease [5], and female, black, Hispanic, or uninsured patients with atrial fibrillation received fewer catheter ablations compared to others with the same comorbidity in the US [6].

A subpopulation of patients with mental illness makes repeated and frequent visits, colloquially labelled as “frequent flyers” [7]. They are thought to visit medical centres frequently and repeatedly with nontypical symptoms, sometimes insisting on their right to receive medical care [8]. In a retrospective observational study on frequent flyers in an emergency department in a rural hospital in Japan, as few as 28 frequent flyers accounted for 5.4% of total 15,343 visits in a year. Of these, 66% of the visits were psychiatric problems with mild symptoms, and 72% of the visits were out of hospital hours, which had obviously been a huge burden for health professionals [9]. In fact, it has been reported that psychiatric patients who make frequent visits to the emergency department are less likely to receive appropriate medical examination [7].

In Japan, the public welfare service provides compensations for all medical costs of the poorer population, which comprises 2,125,317 beneficiaries. Thus, 1.7% of the population in Japan receives free medical care, which costs 4.2% of the total medical expenses in Japan. Free medical care recipients, who are usually socially vulnerable, are also known to visit hospitals frequently with minor complaints to acquire medication or to just interact with hospital staff [10]. For those socially vulnerable populations, playing the sick role as a patient is an easy way to receive compassion from others.

When it comes to the emotional response of professional healthcare providers toward those socially vulnerable populations, few studies have been reported. If the response were unfavourable, it would be against professionalism, and there could be publication bias not to report unprofessional attitude toward socially vulnerable population. Interestingly, Mamede et al. reported unfavourable patients’ behaviours negatively affected physicians’ diagnostic process, leading to diagnostic mistakes [11] and lower likability ratings of physicians toward problematic patients [12].

As for clinical decision making, few studies have been reported about the effect of biasing social background information and the influence of physicians’ emotional reaction toward socially vulnerable population. In the present study, we intend to clarify the impact of patients’ psychiatric or social background information, such as the presence of schizophrenia or being a recipient of free medical care with a history of frequent visits, on physicians’ clinical decision making in terms of conducting further investigation or scheduling a follow-up visit.

## Methods

### Study design

We performed a randomized, controlled multi-centre study on physicians in training at five teaching hospitals. Participants responded to questions pertaining to clinical decision making based on eight clinical vignettes. They were stratified by gender and postgraduate year using a blocked randomization list provided by Sealed Envelope Ltd. 2016 [13]. Subsequently, they were blindly allocated to scenario set 1 or 2 in a counterbalanced way. Half of the eight vignettes presented to scenario set 1 included additional patient information, such as past medical history of schizophrenia or being a recipient of free medical care with a history of frequent visits (biased vignettes). The other half included no additional information (neutral vignettes). Thus, the eight clinical vignettes presented to scenario set 1 consisted of four biased and four neutral clinical vignettes. For scenario set 2, the four biased vignettes presented to scenario set 1 were neutralized, and the four neutral ones were rendered biased by providing additional information. The biased and neutral vignettes presented to scenario set 2 were the opposite of those presented to scenario set 1. Thus, we designed the factor of biasing information to be counterbalanced to minimize the case-dependent effect. Except for the additional information, the clinical vignettes presented to the two scenario sets were identical.

### Participants

The participants were residents in the transitional, internal medicine, or emergency medicine departments, whose postgraduate year were from one to eight at five teaching hospitals: Tokyo Bay Urayasu Ichikawa Medical Centre, Chiba University Hospital, Tsukuba University Hospital, Kyorin University Hospital, and St. Marianna University Hospital. Transitional residents were post graduate year one or two. Senior residents were clinicians who finished a two-year transitional training and engaged in advanced residency program in internal medicine or emergency medicine, whose postgraduate year range from three to eight. We chose residents in internal medicine and emergency medicine who saw patients with a broad range of illnesses in their daily practice. We calculated that the observation of 94 participants in each scenario set would provide 80% power to detect a 20% difference in the effects of additional patient background information (50% vs 30%).

### Materials

The eight clinical vignettes described commonly encountered illnesses, which were pneumonia (case A), pyelonephritis (case B), heart failure (case C), and upper gastrointestinal bleeding (case D), and their common signs and symptoms, which were chest pain (case E), dizziness (case F), syncope (case G), and palpitation (case H). To maximize their validity, we developed case scenarios that described the types of acutely ill patients often encountered in hospitals in Japan. In each vignette, the case presentation was followed by four questions regarding clinical decision making. The first four cases had confirmed diagnoses of pneumonia, pyelonephritis, heart failure, and upper gastrointestinal bleeding, respectively, and each case presentation was followed by four yes or no questions that determined whether a physician would perform or request different diagnostic modalities, interventions, or administration orders. The last four cases showed generic symptoms such as chest pain, dizziness, syncope, and palpitation, and each case presentation was followed by four yes or no questions that determined whether a physician would perform different diagnostic modalities or schedule the next medical follow-up appointment. Content of questions varied among vignettes (Table 1). Clinical decision making questions for four vignettes with a confirmed diagnosis were designed to focus more on decision making after diagnosis, and questions for four vignettes without a diagnosis were designed to focus more on the clinical decision making to make a diagnosis.

**Table 1:** Overview of cases and questions

Case A	*55 year-old man with pneumonia, PORT study Class lll, with or without free medical care access and frequent visit history*	
Q1	Test	Do you order blood culture test? Yes or no?
Q2	Test	Do you order chest CT scan? Yes or no?
Q3	Admission	Do you hospitalize the patient? Yes or no?
Q4	Emergent procedure	Do you perform thoracentesis if there were suspicion of empyema in the chest CT scan? Yes or no?
Case B	*55 year-old man with pyelonephritis, vitals stable, with or without free medical care access and frequent visit history*	
Q1	Test	Do you order blood culture test? Yes or no?
Q2	Test	Do you order abdominal ultrasound exam? Yes or no?
Q3	Admission	Do you hospitalize the patient? Yes or no?
Q4	Emergent procedure	Do you perform urinary tract drainage if there were hydronephrosis with a 15 mm urinary stone? Yes or no?
Case C	*55 year-old man with heart failure and mild pulmonary oedema, vitals stable, with or without schizophrenia and frequent visit history*	
Q1	Test	Do you order arterial blood gas test? Yes or no?
Q2	Treatment	Do you administer furosemide? Yes or no?
Q3	Admission	Do you hospitalize the patient? Yes or no?
Q4	Emergent procedure	Do you administer catecholamine if there were circulatory failure? Yes or no?
Case D	*55 year-old man with upper gastrointestinal bleeding, Blatchford Score 1, vitals stable, with or without schizophrenia*	
Q1	Test	Do you order blood test? Yes or no?
Q2	Treatment	Do you perform emergent esophagogastroduodenoscopy? Yes or no?
Q3	Admission	Do you hospitalize the patient? Yes or no?
Q4	Emergent procedure	Do you perform red blood cell transfusion if haemoglobin were 6.5 g/dL? Yes or no?
Case E	*55 year-old man with atypical chest pain, with or without schizophrenia and frequent visit history*	
Q1	Test	Do you order electrocardiogram? Yes or no?
Q2	Test	Do you order troponin test? Yes or no?
Q3	Test	Do you order d-dimer test? Yes or no?
Q4	Follow-up	Do you schedule the next medical follow-up visit? Yes or no?
Case F	*55 year-old man with gradual onset continuous dizziness, with or without schizophrenia and frequent visit history*	
Q1	Test	Do you order blood test? Yes or no?
Q2	Test	Do you order head CT scan? Yes or no?
Q3	Test	Do you order head MRI scan? Yes or no?
Q4	Follow-up	Do you schedule the next medical follow-up visit? Yes or no?
Case G	*55 year-old man with transient loss of consciousness, spontaneous recovery, with or without free medical care access*	
Q1	Test	Do you order electrocardiogram? Yes or no?
Q2	Test	Do you order head CT scan? Yes or no?
Q3	Treatment	Do you perform tetanus vaccination? Yes or no?
Q4	Follow-up	Do you schedule the next medical follow-up visit? Yes or no?
Case H	*55 year-old man with palpitation, heart rate 90/min regular, with or without free medical care access and frequent visit history*	
Q1	Test	Do you order electrocardiogram? Yes or no?
Q2	Test	Do you order troponin test? Yes or no?
Q3	Test	Do you order thyroid function test? Yes or no?
Q4	Follow-up	Do you schedule the next medical follow-up visit? Yes or no?

PORT study: Pneumonia Patient Outcomes Research Team study

The clinical cases were prepared, based on real patients, by three board-certified emergency physicians (TS, HF, and MM) and three board-certified internists (MI, EH, and KS). The three internists and three emergency physicians confirmed that each question was valid and understandable. The preliminary study was conducted among physicians and was modified according to their feedback. The complete set of vignettes is available on request.

### Procedure

The participants were asked to read the eight clinical vignettes and answer four clinical decision-making questions for each vignette, without knowing the objectives of the study beforehand. These questions concerning clinical management choice were yes or no questions. The three board-certified emergency physicians (TS, HF, and MM) and the three board-certified internists (MI, EH, and KS) discussed and defined which management choices were appropriate, partially appropriate, or inappropriate (scored as 1, 0.5, or 0 points, respectively) for each clinical vignette to calculate the management accuracy score. When these board-certified clinicians did not reach a consensus, the answers to these questions were categorized as partially appropriate choices. To measure the influence of the participants’ emotional impression about the patient from the case presentation, participants used a five-point Likert scale to assess how favourable their perception of the patient was after answering questions. The background information of the participants was collected using a questionnaire on age, gender, postgraduate year, specialty, psychiatry rotation experience, completing a night shift directly prior to responding to the questionnaire, and emotional intelligence. Emotional intelligence was assessed by the emotional quotient (EQ), measured using four factors (well-being, self-control, emotionality, and sociability), and the total score on the Trait Emotional Intelligence Questionnaire-Short Form (TEIQue-SF) [14], which has been validated in multiple languages and used in scientific research.

### Data analyses

The multiple imputation method was used to address missing data. Continuous variables were expressed as means (standard deviation, SD) or medians (interquartile range, IQR), as appropriate, and discrete variables were summarized as percentages.

For analysis of participants’ background variables, continuous variables were analysed and compared using the Mann-Whitney U test, and dichotomous variables were analysed and compared using the χ^2^ test.

There are two types of allocation of case scenarios for participants in scenario set 1 or 2 in a counterbalanced way in terms of additional background information. In scenario set 1, one participant is asked to answer the question regarding cases B and H with biasing information of free access to medical care (free medical care recipients condition), cases D and F with biasing information of past schizophrenia history (schizophrenia condition), cases A and G with no biasing patient background information (no bias condition 1, a counterpart of free medical care recipients condition), and cases C and E with no biasing patient background information (no bias condition 2, a counterpart of schizophrenia condition). In scenario set 2, the other participant is asked to answer the question regarding cases A and G with biasing information of free access to medical care (free medical care recipients condition), cases C and E with biasing information of past schizophrenia history (schizophrenia condition), cases B and H with no biasing patient background information (no bias condition 1), and cases D and F with no biasing patient background information (no bias condition 2). Our primary analysis is the repeated-measures ANOVA on the mean management accuracy score with patient background information as a within-subject factor (no biasing information, free medical care recipients, or medical history of schizophrenia), followed by post hoc pairwise multiple comparison test, Sidak test.

Cases A, B, C, and D contained the confirmed diagnosis, and cases E, F, G, and H comprised only symptoms without diagnosis. To assess the influence of the factor of whether a diagnosis is presented or not, secondary analyses were conducted for four vignettes with a diagnosis (cases A, B, C, and D) and four vignettes without a diagnosis (cases E, F, G, and H) using repeated-measures ANOVA on the mean management accuracy score with patient background information as a within-subject factor (no biasing information, free medical care recipients, or medical history of schizophrenia), followed by post hoc pairwise multiple comparison test, Sidak test.

Further, a repeated-measures ANOVA was conducted for the ratings for patient likability in each condition (no biasing information, free medical care recipients, or medical history of schizophrenia). Lastly, Likert scale 1–2 was categorized as unfavourable impression, scale 3 was categorized as neutral impression, and scale 4–5 was categorized as favourable impression toward patients. The number of physicians who answered yes to spend additional medical resources for each question for each vignette was analysed and compared using the χ^2^ test among unfavourable, neutral, or favourable patient impression.

JMP® 13 (SAS Institute Inc., Cary, NC, USA) and STATA® 16 (StataCorp LLC, College Station, TX, USA) were used to perform the statistical analysis. *P* values of less than 0.05 were considered statistically significant.

## Results

### Participants

**Fig. 1:**
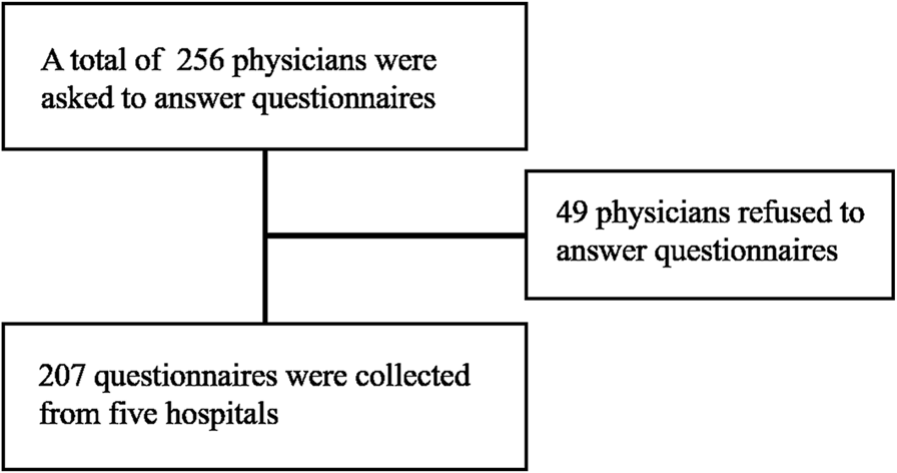
Questionnaire collection

A total of 207 questionnaires were collected (response rate 81%) from the five hospitals (Fig. 1). Five out of 207 questionnaires were incomplete, and missing data were addressed using the multiple imputation method. There were no significant differences in characteristics between the participants in scenario sets 1 and 2 (Table 2). The median (IQR) age was 28 (26–29) years. The median (IQR) number of postgraduate years was 2 (1–3) years. Additionally, 71% (146/207) of the participants were men, 70% (144/207) were transitional residents, 22% (46/207) were internal medicine residents, and 8% (17/207) were emergency medicine residents. Further, 41% (84/207) of the participants had experienced a psychiatry rotation in their residency program, and 18% (38/207) completed the present questionnaires immediately after completing a night shift.

**Table 2:** Characteristics of the participants

	Scenario set 1 (*n* = 102)	Scenario set 2 (*n* = 105)
Age (years), median (IQR)	28 (26–29)	28 (26–29)
Men, No. (%)	71 (70%)	75 (71%)
Postgraduate year, median (IQR)	2 (1–3)	2 (1–3)
Transitional resident, No. (%)	70 (71%)	69 (67%)
Senior resident, No. (%)	29 (29%)	34 (33%)
Specialty		
Transitional resident, No. (%)	73 (71%)	71 (67%)
Internal medicine, No. (%)	21 (21%)	25 (24%)
Emergency medicine, No. (%)	8 (8%)	9 (9%)
Hospital		
Tokyo Bay Medical Centre, No. (%)	18 (18%)	21 (20%)
Chiba University, No. (%)	14 (14%)	20 (19%)
Tsukuba University, No. (%)	17 (17%)	15 (14%)
Kyorin University, No. (%)	32 (31%)	28 (27%)
St. Marianna University, No. (%)	21 (20%)	21 (20%)
Rotation in psychiatry, No. (%)	39 (38%)	45 (43%)
Just after night shift, No. (%)	18 (18%)	20 (19%)
Total EQ score, mean (SD)	4.2 (0.63)	4.2 (0.63)
Well-being, mean (SD)	4.5 (1.0)	4.5 (0.90)
Self-control, mean (SD)	4.0 (0.84)	4.0 (0.78)
Emotionality, mean (SD)	4.4 (0.71)	4.3 (0.78)
Sociability, mean (SD)	4.0 (0.83)	3.9 (0.77)

*IQR* interquartile range, *EQ* emotional quotient, *SD* standard deviation

### Clinical management accuracy score

**Fig. 2:**
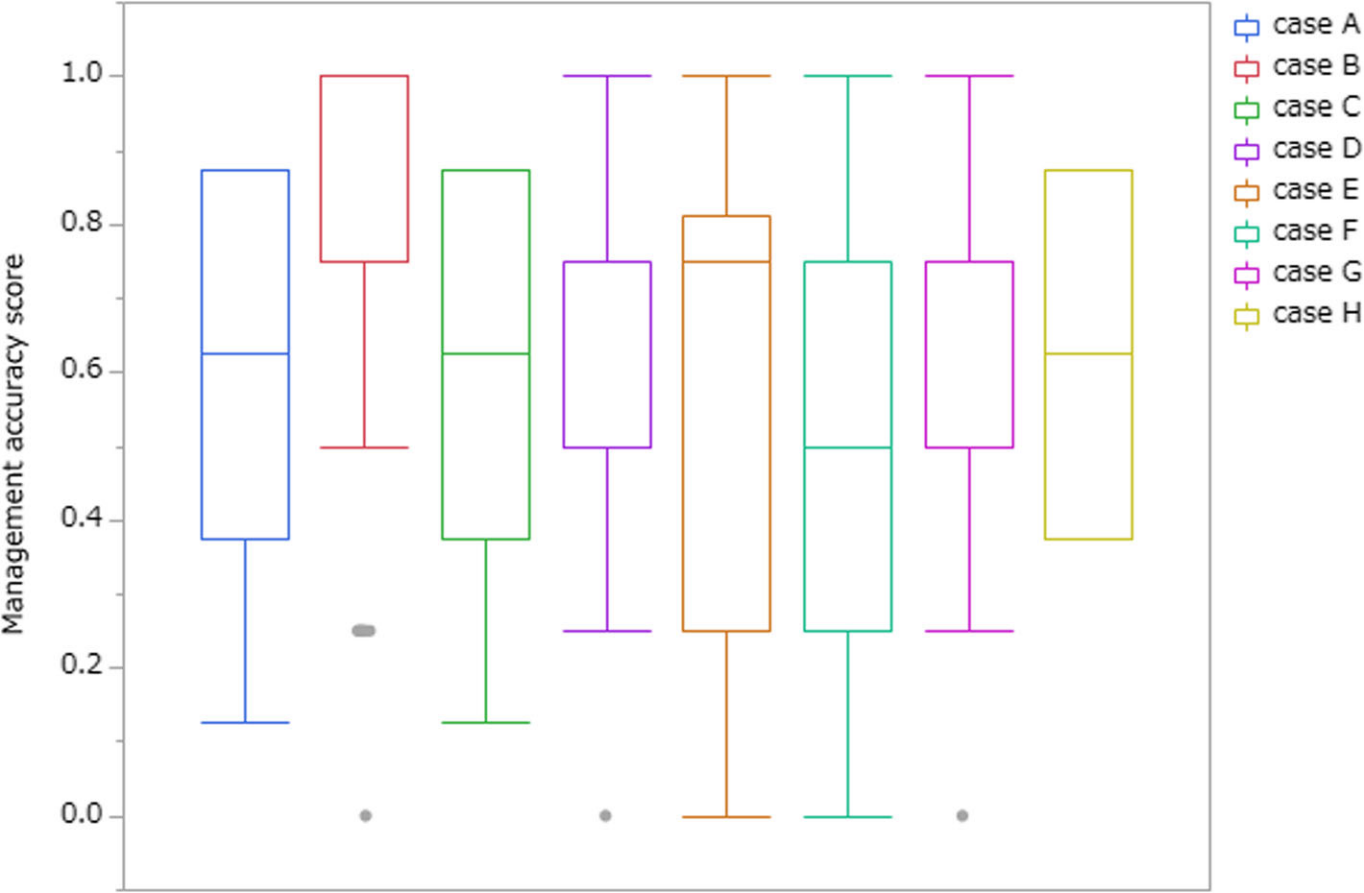
Mean management accuracy score of cases A to H

**Fig. 3:**
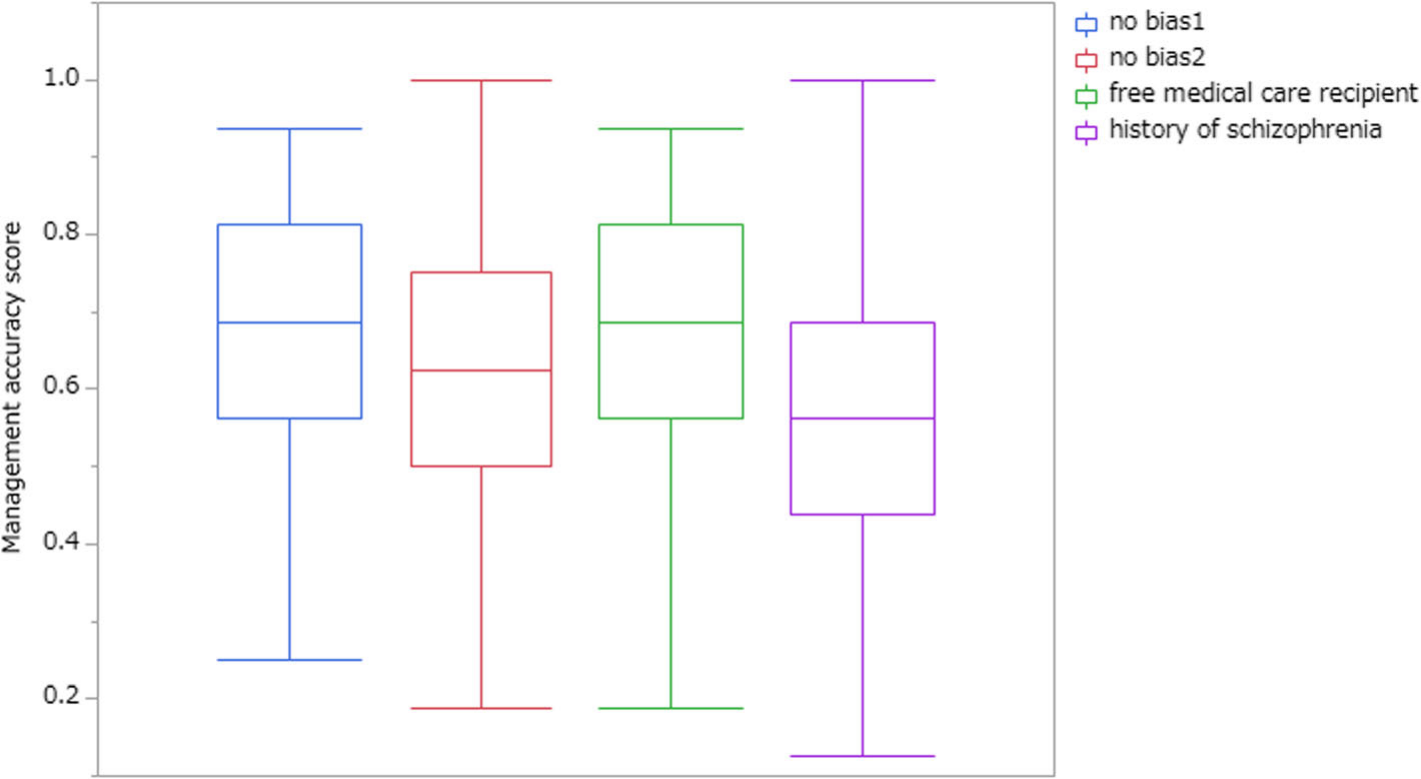
Mean management accuracy score by additional background information. No bias condition 1, a counterpart of free medical care recipients condition. No bias condition 2, a counterpart of schizophrenia condition

Management accuracy score varied from case to case (Fig. 2). Figure 3 shows management accuracy score of each condition (no biasing information, free medical care recipients, or history of schizophrenia). Repeated-measures ANOVA showed that additional background information had an influence on mean accuracy score (F(7, 206) = 13.84, *p* <  0.001, partial η2 = 0.063). Post hoc pairwise multiple comparison test, Sidak test, showed a significant difference between schizophrenia and no bias condition (*p* <  0.05).

Repeated-measures ANOVA for vignettes with a diagnosis in dataset of cases A, B, C, and D revealed that additional background information (no bias, free medical care recipient, or history of schizophrenia) had an influence on mean accuracy score (F(3, 206) = 16.24, p <  0.001, partial η2 = 0.074). However, post hoc pairwise multiple comparison test, Sidak test, did not show significant difference between types of additional information. Repeated-measures ANOVA for vignettes without a diagnosis in dataset of cases E, F, G, and H demonstrated that additional information had an influence on mean accuracy score (F(3, 206) = 12.00, *p* <  0.001, partial η2 = 0.055). Post hoc pairwise multiple comparison test, Sidak test, exhibited significant difference between schizophrenia and no bias condition (*p* <  0.05).

### Likability ratings for patients

**Fig. 4:**
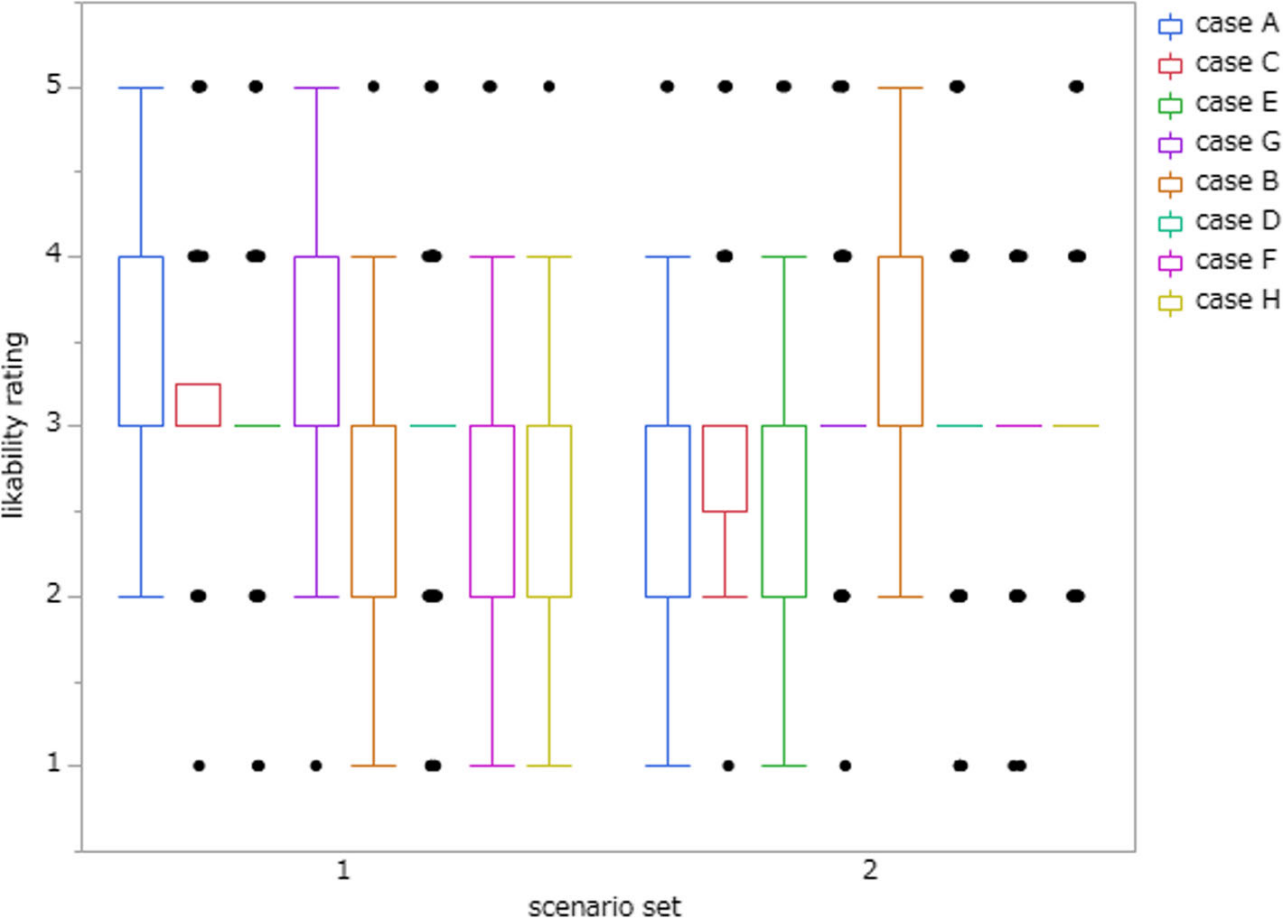
Likability rating in cases A to H. AG and CE: no biasing information, BH: free medical care recipients, DF: schizophrenia in scenario set 1. AG: free medical care recipients, CE: schizophrenia, BH and DF: no biasing information in scenario set 2

**Fig. 5:**
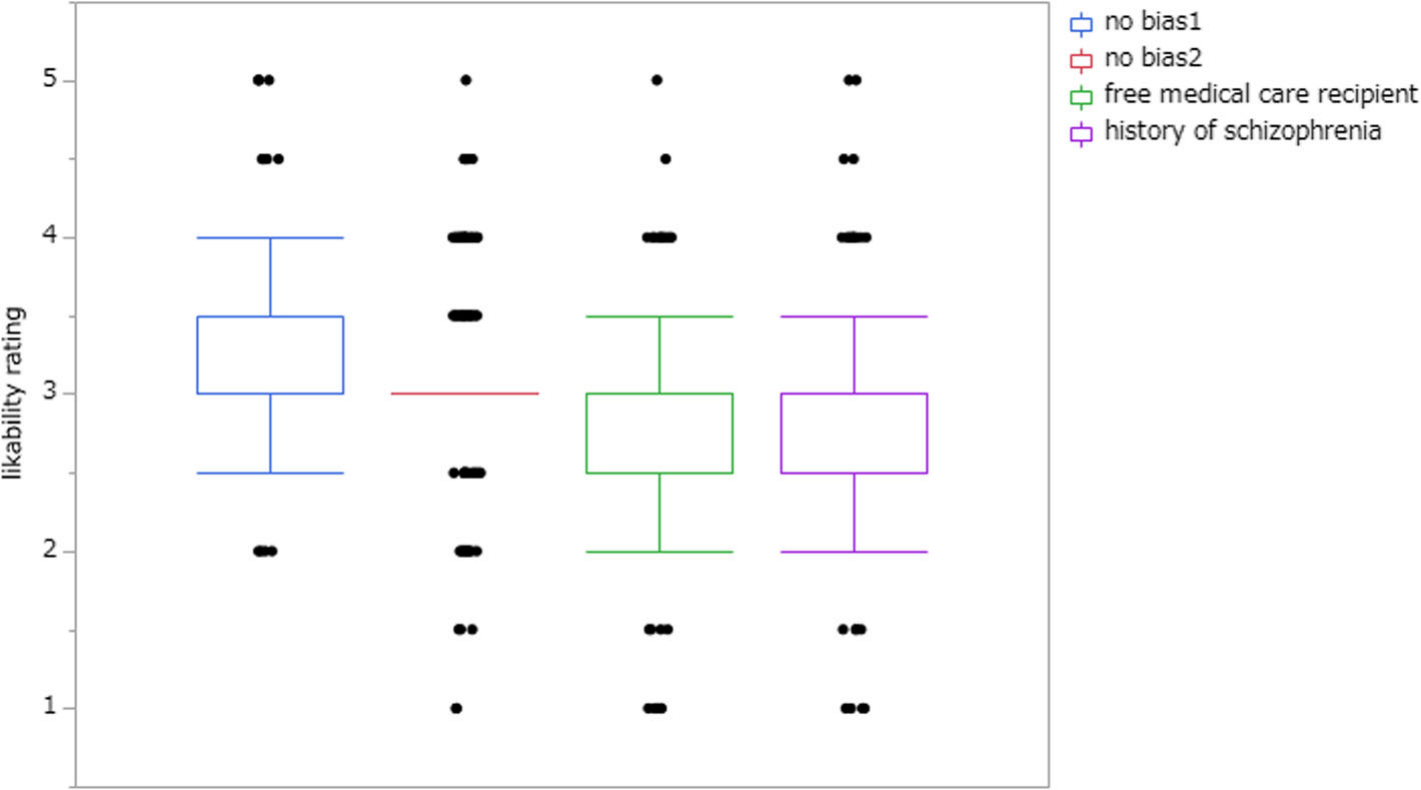
Likability rating by additional background information. No bias condition 1, a counterpart of free medical care recipients condition. No bias condition 2, a counterpart of schizophrenia condition

Likability ratings for patients in each case are illustrated in Fig. 4. Figure 5 shows likability ratings of each condition (no biasing information, free medical care recipients, or history of schizophrenia). Repeated-measures ANOVA proved that additional background information had an influence on likability ratings (F(7, 206) = 21.08, *p* <  0.001, partial η2 = 0.378). Post hoc pairwise multiple comparison test, Sidak test, showed a significant difference between schizophrenia and no bias condition (*p* <  0.001) and between free medical care recipients and no bias condition (*p* <  0.001). As the patients’ impression was favourable in general, the number of physicians who would spend additional medical resources, such as by recommending further tests or treatment, by admitting the patient to hospital, or by scheduling a follow-up visit, increased (Table 3).

**Table 3:** Number of physicians (%) who would spend additional medical resources

Patient impression (Likert scale)	Unfavourable (1–2)	Neutral (3)	Favourable (4–5)	*p* value*
Case A				
Q1	24 (72%)	106 (80%)	33 (89%)	0.20
Q2	21 (64%)	79 (60%)	25 (68%)	0.67
Q3	15 (45%)	79 (60%)	28 (76%)	< 0.05
Q4	22 (67%)	79 (60%)	26 (70%)	0.45
Case B				
Q1	36 (84%)	106 (89%)	39 (98%)	0.08
Q2	38 (88%)	113 (95%)	37 (93%)	0.37
Q3	33 (77%)	96 (81%)	34 (85%)	0.63
Q4	27 (63%)	63 (53%)	29 (73%)	0.07
Case C				
Q1	20 (63%)	102 (79%)	34 (83%)	0.10
Q2	8 (25%)	65 (50%)	20 (49%)	< 0.05
Q3	15 (47%)	95 (74%)	26 (63%)	< 0.05
Q4	17 (53%)	64 (50%)	24 (59%)	0.60
Case D				
Q1	35 (97%)	129 (97%)	33 (100%)	0.40
Q2	2 (6%)	24 (18%)	12 (36%)	< 0.01
Q3	11 (31%)	62 (47%)	15 (45%)	0.21
Q4	29 (81%)	128 (96%)	28 (85%)	< 0.01
Case E				
Q1	35 (95%)	127 (94%)	29 (97%)	0.84
Q2	17 (46%)	85 (63%)	24 (80%)	< 0.05
Q3	10 (27%)	58 (43%)	16 (53%)	0.08
Q4	10 (27%)	72 (53%)	16 (53%)	< 0.05
Case F				
Q1	32 (58%)	94 (72%)	13 (81%)	0.10
Q2	37 (67%)	97 (74%)	13 (81%)	0.46
Q3	11 (20%)	31 (24%)	5 (31%)	0.64
Q4	19 (35%)	76 (58%)	11 (69%)	< 0.01
Case G				
Q1	22 (92%)	130 (98%)	45 (98%)	0.26
Q2	21 (88%)	128 (97%)	43 (93%)	0.17
Q3	8 (33%)	38 (29%)	6 (13%)	0.06
Q4	14 (58%)	97 (73%)	29 (63%)	0.20
Case H				
Q1	58 (97%)	124 (100%)	18 (100%)	0.09
Q2	14 (23%)	47 (38%)	10 (56%)	< 0.05
Q3	28 (47%)	84 (68%)	13 (72%)	< 0.05
Q4	16 (27%)	59 (48%)	15 (83%)	< 0.01

*: the statistical analysis was performed using the χ^2^ test

## Discussion

Our study revealed that the physicians’ clinical decision making was influenced by patients’ background information, such as past medical history of schizophrenia. This biasing background information elicited negative affective responses in physicians, resulting in lower ratings of patient likability, which in turn were related to the allocation of fewer medical resources by physicians.

To the best of our knowledge, ours is the first study to show that biasing information, such as past medical history of severe mental illness with a history of frequent visits to hospitals, elicits physicians’ biases, thus affecting their decision-making processes. The biasing information particularly influenced the total score of management accuracy when the diagnosis was not confirmed. In our study, patient likability rating was a main-effect modifier in the decision making process of physicians. It has been widely acknowledged that community and healthcare professionals hold negative feelings toward patients with schizophrenia [15, 16]. Although most psychiatric patients are not violent, it is retrospectively reported that schizophrenia is related to aggressive behaviour toward people or property in an emergency department setting [17]. Table 3 indicates that the likability rating assigned by the participants for patients with schizophrenia influenced their clinical decision making.

It has been shown that the emotional state of physicians leads to biases in clinical reasoning and judgment [18]. Furthermore, problematic patients who behave disruptively tend to evoke physicians’ negative emotions and induce diagnostic errors [11, 12]. Medical students can also find it challenging to prevent their emotions from interfering with their clinical judgments [19]. While these studies indicate that healthcare providers should be aware of their emotional state and avoid bias in their judgements, little is known about what kind of information can lead to such a bias and to what extent.

Our study indicates that not only clinical reasoning and diagnosis, but also clinical decision making about whether to order a diagnostic test or schedule a follow-up visit could be impeded by the negative feelings of the providers toward patients with schizophrenia with a history of frequent visits. The term “frequent visit” might induce a negative emotional reaction from novice physicians. In our study, most of the participants were transitional interns in postgraduate year one or two, so they might be susceptible to negative impressions of frequent visitors. This, in turn, may explain their lower inclination to schedule a follow-up visit. However, it is noteworthy that the information about a patient’s frequent visit history should be carefully interpreted because it may imply serious anxiety or, sometimes, serious biological health problems.

Our study indicated physicians made more mistakes on clinical decision making in patients with schizophrenia. In daily outpatient practice in the internal medicine clinic and emergency department, physicians tend to feel stressed when dealing with a specific population of patients, such as those with somatoform disorder, substance abuse disorder, or anxiety disorder. In future studies, we should elucidate the extent of interference of physicians’ emotions toward these populations and clinical decision making, with the aim of improving bias-free clinical practice.

### Limitations of the study

This study has several potential limitations. First, the study was conducted by the block randomization method using data from five teaching hospitals. Because of the potential variation of the data from each hospital, to account for outcome clustering within the participating physicians in each hospital level, we performed a logistic regression analysis with generalized estimating equations. In this additional analysis, we confirmed the same results as in the main analysis, which were that the biasing information led physicians to avoid certain diagnostic tests or to schedule a follow-up visit for patients with schizophrenia or free medical care recipients.

Second, the cases containing biasing information were presented through a basic questionnaire in the present study. The key to using clinical vignettes was to identify the impact of the biasing information on the participants’ impressions about the patient. Simulated patients can easily influence participants’ affect, and this has been utilized successfully in medical education. Recently, scripted video vignettes have been used for medical communication and medical education to appeal to participants’ feelings [20]. However, simulated patients and video vignettes are resource-intensive methods to implement in clinical studies. Nevertheless, our paper-based case presentation successfully addressed the objective of our study, by demonstrating that including biasing information induced a decrease in patient likability rating.

Third, in some clinical decision-making questions about conducting an additional diagnostic test, such as a blood test or an electrocardiogram, more than 94% of the participants chose to order such inexpensive and noninvasive tests. Therefore, the power to detect the influence of biasing information was limited in some questions. In future studies, a more elaborate study design will be needed to tailor clinical decision-making questions to physicians’ clinical experience.

## Conclusions

This study showed physicians’ tendencies to avoid running certain diagnostic tests or scheduling a follow-up visit for patients with past medical history of schizophrenia especially when the diagnosis was not confirmed. Additionally, it indicated that the favourable or unfavourable impressions about the patient had a great influence on physicians’ clinical decision making. This background information should not bias clinical decision making, and physicians should make an effort to be free from bias. Our study clarifies this bias and recommends that physicians try to avoid being influenced by medically unrelated information and their impression about the patient.

## Data Availability

The raw dataset supporting the conclusions of this article is available from the corresponding author upon request.

## Abbreviations

CI: Confidence intervals
EQ: Emotional quotient
IQR: Interquartile range
SD: Standard deviation
TEIQue-SF: Trait Emotional Intelligence Questionnaire-Short Form
